# Supplementary material for: Lenvatinib Exacerbates the Decrease in Skeletal Muscle Mass in Patients with Hepatocellular Carcinoma, Whereas Atezolizumab Plus Bevacizumab Does Not
Source: Cancers (Basel). 2024 Jan 19;16(2):442. doi: 10.3390/cancers16020442 (PMC10814020; doi:10.3390/cancers16020442)
Supplement: Supplementary file 1 [file cancers-16-00442-s001.zip › Table S1.pdf]

Table S1. The pre-, combination, and post-treatment results of the enrolled patients

	AB group (n = 37)			LEN group (n = 57)		
	Pre-treatment	Combination treatment	Post-treatment	Pre-treatment	Combination treatment	Post-treatment
Any treatments	33 (89.2%)	4 (10.8%)	15 (40.5%)	51 (89.5%)	14 (24.6%)	28 (49.1%)
Hepatectomy	20	0	0	23	0	0
RFA	17	1	0	9	2	1
TACE	21	3	5	35	12	17
Radiation therapy	6	0	2	16	1	2
Other chemotherapy	8	0	14	17	0	15

RFA, radiofrequency ablation; TACE, transcatheter arterial chemo embolization;
