# Supplementary material for: Lenvatinib Exacerbates the Decrease in Skeletal Muscle Mass in Patients with Hepatocellular Carcinoma, Whereas Atezolizumab Plus Bevacizumab Does Not
Source: Cancers (Basel). 2024 Jan 19;16(2):442. doi: 10.3390/cancers16020442 (PMC10814020; doi:10.3390/cancers16020442)
Supplement: Supplementary file 1 [file cancers-16-00442-s001.zip › Table S2.pdf]

Table S2. Changes in body composition during initial treatment and the introduction of AB or LEN treatment

	AB group (n = 37)			LEN group (n = 57)		
	Initial treatment	Introduction of AB	*p* value	Initial treatment	Introduction of LEN	*p* value
SMI (cm^2/m^2)	43.2 (37.8–49.0)	42.9 (36.4–47.7)	0.777	45.0 (39.8–51.4)	46.5 (40.6–50.8)	0.931
SATI (cm^2/m^2)	50.0 (26.2–73.2)	42.8 (30.4–53.8)	0.119	47.1 (26.0–59.0)	52.1 (27.9–67.6)	0.164
VATI (cm^2/m^2)	44.7 (27.6–60.8)	54.7 (36.1–67.2)	0.002	47.0 (27.4–65.4)	48.8 (29.0–64.2)	0.891

Continuous covariates are presented as medians (interquartile range). AB, atezolizumab plus bevacizumab; LEN, Lenvatinib; SMI, skeletal muscle index; SATI, subcutaneous adipose tissue index; VATI, visceral adipose tissue index;
