# Supplementary figures and images for: Lenvatinib Exacerbates the Decrease in Skeletal Muscle Mass in Patients with Hepatocellular Carcinoma, Whereas Atezolizumab Plus Bevacizumab Does Not
Source: Cancers (Basel). 2024 Jan 19;16(2):442. doi: 10.3390/cancers16020442 (PMC10814020; doi:10.3390/cancers16020442)

Figure S1

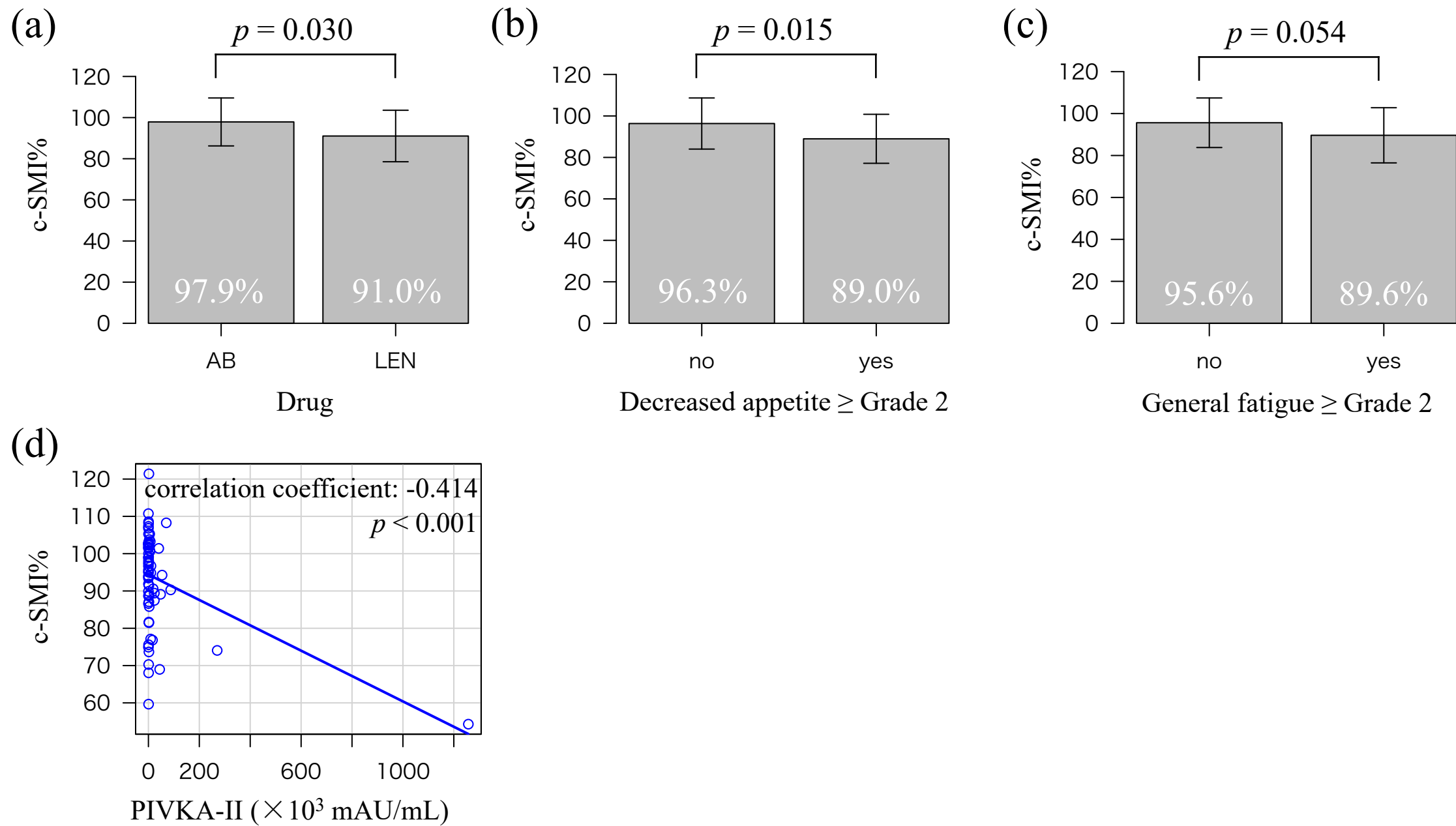

Supplement: Supplementary file 1 [file cancers-16-00442-s001.zip › Figure S1.pdf]
